# Supplementary material for: Risk assessment for airborne disease transmission by poly-pathogen aerosols
Source: PLoS One. 2021 Apr 8;16(4):e0248004. doi: 10.1371/journal.pone.0248004 (PMC8031403; doi:10.1371/journal.pone.0248004)
Supplement: S2 Appendix — Checking the recursive analytical solution against solving the system of equations in Eq (47) numerically. (PDF) [file pone.0248004.s002.pdf]

Risk assessment for airborne disease transmission by poly-pathogen aerosols

Freja Nordsiek[1], Eberhard Bodenschatz[1,2,3*], Gholamhossein Bagheri[1],

1 Max Planck Institute for Dynamics and Self-Organization (MPIDS), Göttingen, Niedersachsen, Germany
2 Institute for Dynamics of Complex Systems, University of Göttingen, Göttingen, Niedersachsen, Germany
3 Laboratory of Atomic and Solid State Physics and Sibley School of Mechanical and Aerospace Engineering, Cornell University, Ithaca, New York, USA

* lfpn-office@ds.mpg.de

S2 Appendix. Checking Analytical Solution Against Numerical Solution

The recursive analytical solution for $\vec{n}$ will be compared against a numerical solution. Lets consider, for some fixed d_0, the following case.

$$\begin{aligned}
M_c &= 10 \\
\alpha &= \frac{3}{2} \quad [\mathrm{T}]^{-1} \\
\beta &= 50 + 20k \quad [\mathrm{L}]^{-4}\,[\mathrm{T}]^{-1} \\
\gamma &= \frac{1}{10} \quad [\mathrm{T}]^{-1} \\
n_{0,k} &= \left|\frac{M_c}{2} - |4-k|\right|^4 \quad [\mathrm{L}]^{-4}
\end{aligned}$$

where [T] is the unit of time and [L] is the unit of length. This example starts with both some $n_{0,k} < n_{\infty,k}$ and some $n_{0,k} > n_{\infty,k}$ and a source whose strength increases with k. The system was solved from $t = t_0 = 0$ [T] to $t = 100$ [T] in steps of 10^{-2} [T] for the analytical solution and 10^{-3} [T] for the numerical solution. The system of ODEs was solved numerically using the standard Runge-Kutta 4 in IEEE 754 `binary64` floating point (commonly known as `float64` or double precision). The small time step was chosen in order to check that the differences between the two solutions are small. The analytical solution to the concentration densities $n_k(d_0, t)$ over time is shown in the left panel in Fig 1, along with the normalized residual between the analytical and numerical solutions (absolute value of the difference divided by the analytical solution) in the right panel. The concentration densities decay or grow from $\vec{n}_0$ towards $\vec{n}_\infty$ as we expect. The differences between the analytical and numerical calculations are small (less than 10^{-12}); sometimes reaching the smallest relative differences that can be represented in IEEE 754 `binary64` numbers with their 53 bit mantissas [1], which are 2.2×10^{-16} (numerical bigger than analytical by a fraction of 2^{-52}) and 1.1×10^{-16} (numerical smaller than analytical by a fraction 2^{-53}).

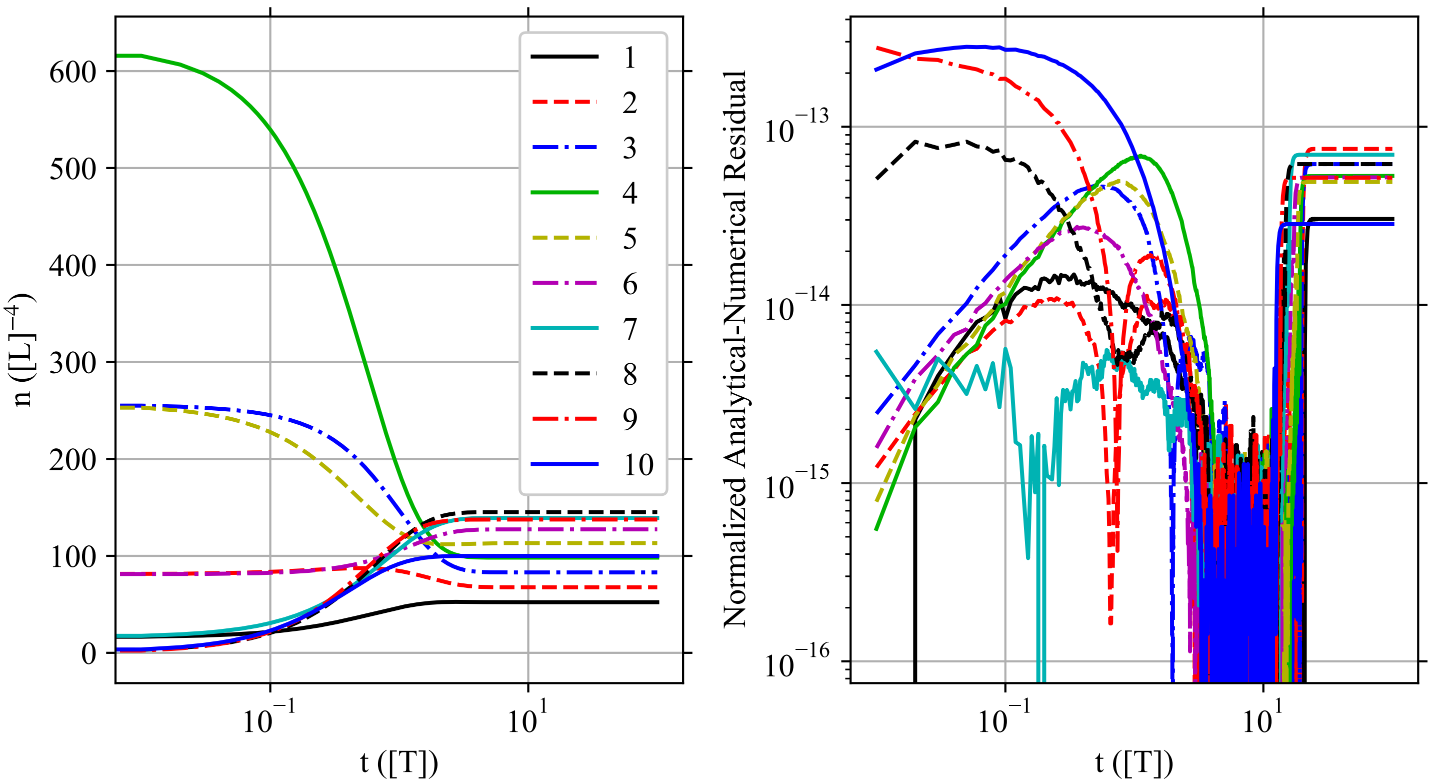

Fig 1. Numerical Validation of Analytical Solution. Comparison of the analytical and numerical solutions of $\vec{n}$ for one particular case and d_0. (Left) Analytical solution (recursive form) to the infectious aerosol concentration density $n_k(t)$ over time, and (Right) the normalized residual between the analytical and numerical solutions $(|n_{k,analytical} - n_{k,numerical}| / n_{k,analytical}$ over all time steps except t_0 where some $n_{0,k}$ are zero. Each k is drawn as a separate line, labeled by the value of k. Both panels share the same legend, which is in the Left panel. The numerical solution was done by Runge-Kutta 4 with a time step of 10^{-3} [T] using IEEE 754 `binary64` arithmetic.

References

1. IEEE Floating-Point Working Group. IEEE Standard for Floating-Point Arithmetic. IEEE Std 754-2019 (Revision of IEEE 754-2008). 2019; p. 1–84. doi:10.1109/IEEESTD.2019.8766229.
