# Supplementary figures and images for: Risk assessment for airborne disease transmission by poly-pathogen aerosols
Source: PLoS One. 2021 Apr 8;16(4):e0248004. doi: 10.1371/journal.pone.0248004 (PMC8031403; doi:10.1371/journal.pone.0248004)

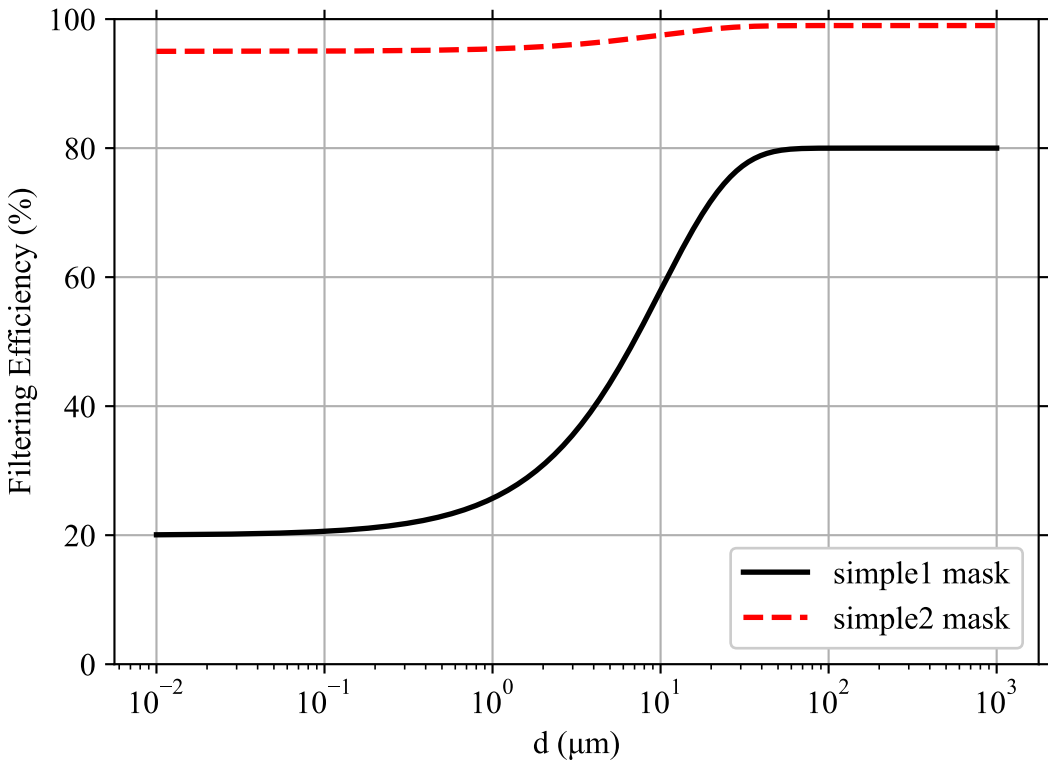

Supplement: S1 Fig — The filtering efficiencies of the simple1 and simple2 masks from the example, whose functional forms are given by Eq (81), as a function of the diameter. (PDF) [file pone.0248004.s006.pdf]

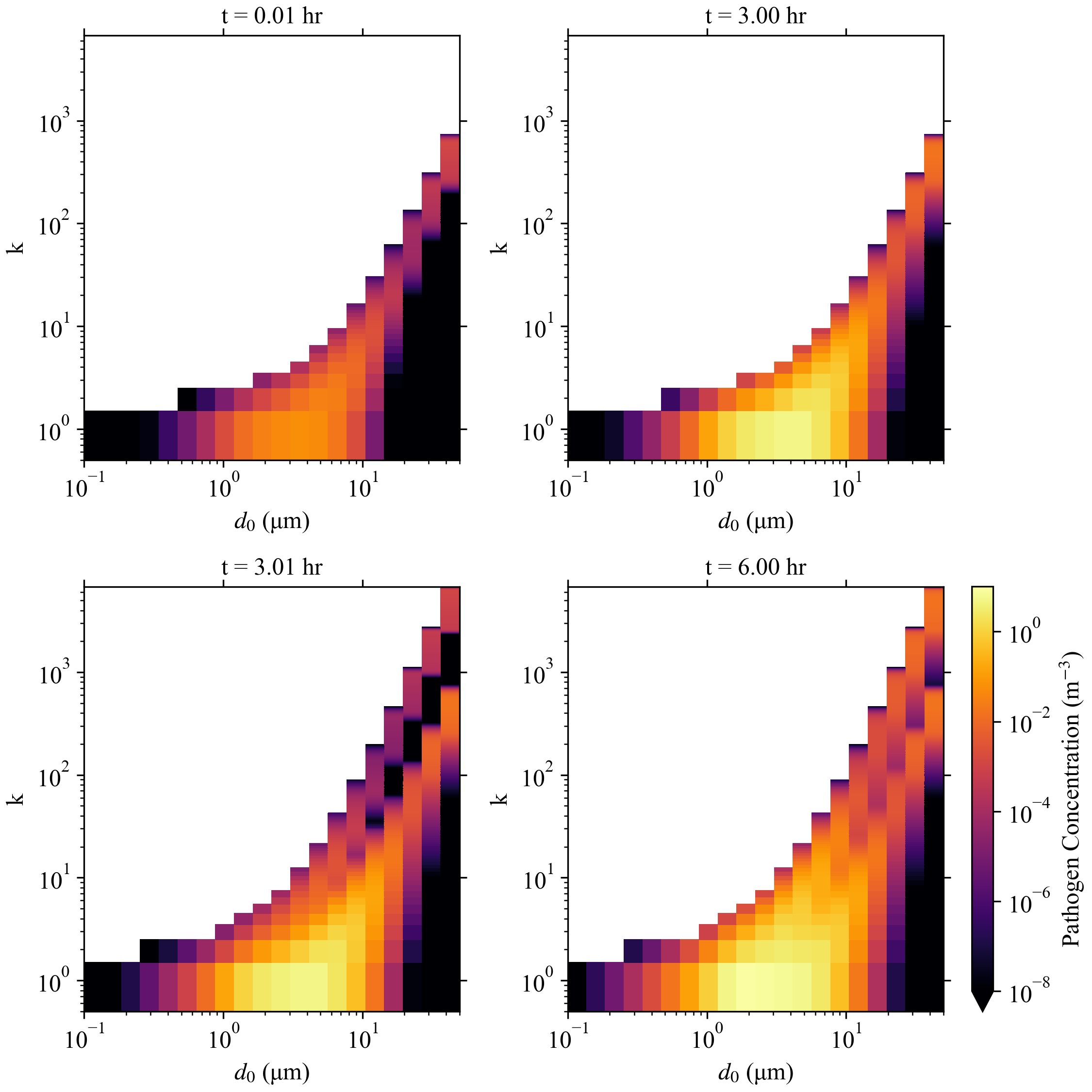

Supplement: S2 Fig — The pathogen concentration in the room as a function of d0 and k, denoted by color, at four different times (listed in the title of each panel) in the example situation. They are (Top-Left) right after the beginning of Stage 1, (Top-Right) at the end of Stage 1, (Bottom-Left) right after the beginning of Stage 2, and (Bottom-Right) at the end of Stage 2. All four panels share the same colorbar, which is in the bottom-right panel. (PNG) [file pone.0248004.s007.png]

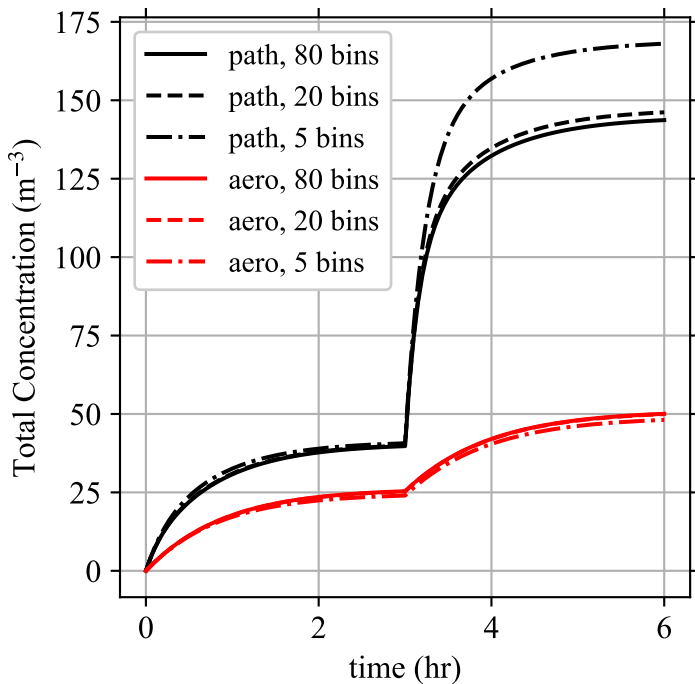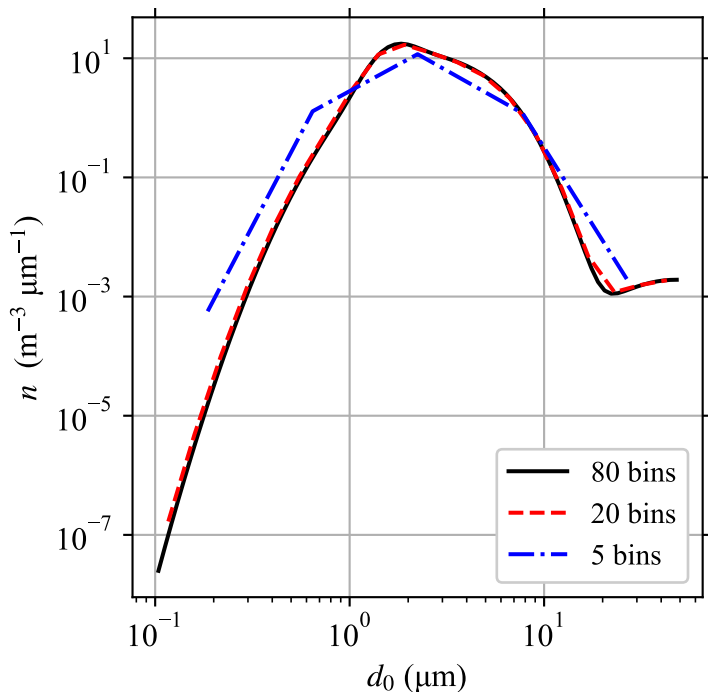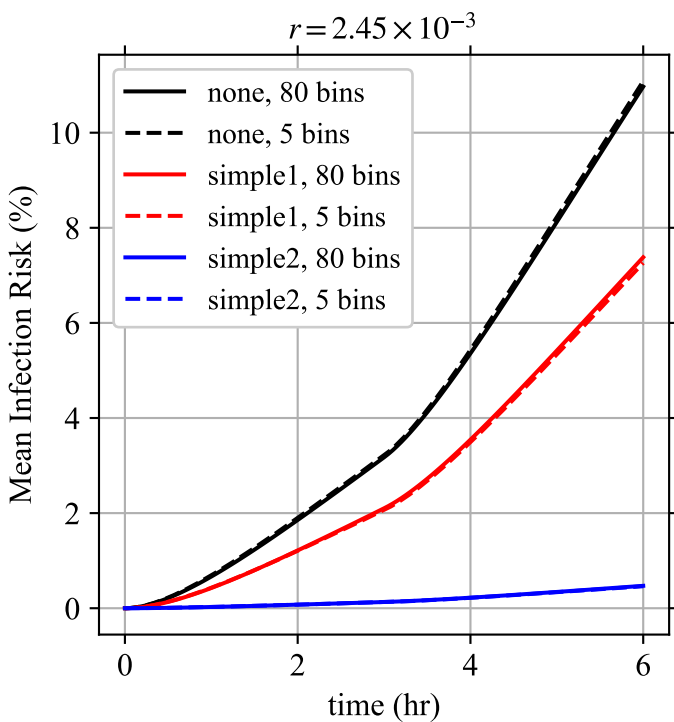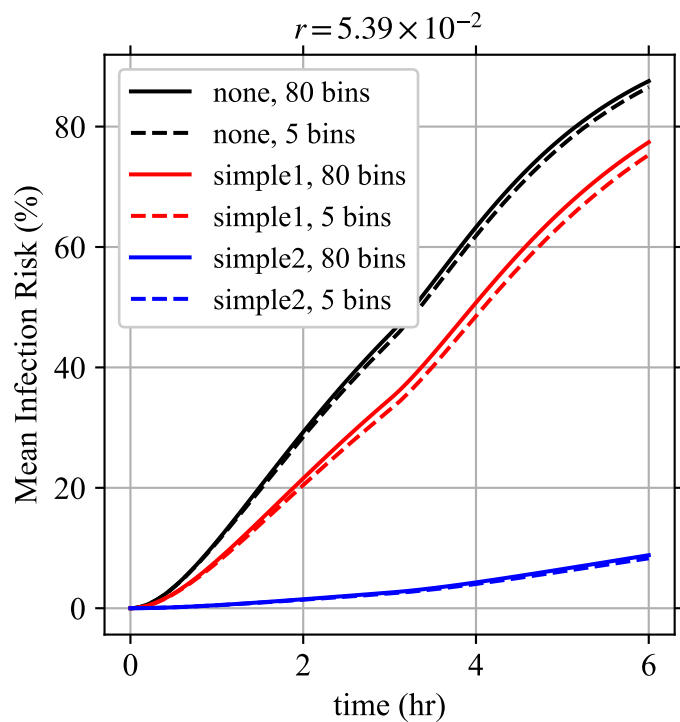

Supplement: S3 Fig — Version of Fig 3, but comparing the model solution for the example situation for 5, 20, and 80 bins. (Top-Left) The total pathogen and infectious aerosol concentrations over time for each number of diameter bins used to solve the model. (Top-Right) The infectious aerosol concentration densities as a function of d0 at t = 6 hr for each number of bins. (Bottom-Left, Bottom-Right) The mean infection risk RE for the susceptible individuals based on the mask they are wearing (none, simple1, or simple2) for each number of bins using (Bottom-Left) r = 2.45 × 10^−3^ and (Bottom-Right) r = 5.39 × 10^−2^. (PDF) [file pone.0248004.s008.pdf]
