# Supplementary material for: Risk assessment for airborne disease transmission by poly-pathogen aerosols
Source: PLoS One. 2021 Apr 8;16(4):e0248004. doi: 10.1371/journal.pone.0248004 (PMC8031403; doi:10.1371/journal.pone.0248004)
Supplement: Attachment — Submitted filename: response_to_reviewers.pdf [file pone.0248004.s009.pdf]

Response to Reviewers: Risk assessment for airborne disease transmission by poly-pathogen aerosols

Freja Nordsiek, Eberhard Bodenschatz*, and Gholamhossein Bagheri

February 15, 2021

1 Introduction

Thank you for taking the time to review our manuscript and give comments. In this document, we will first describe how changes are marked in the revised manuscript with tracked changes. Then, we will respond to your comments one by one and describe changes we have made. And then we will describe changes to the manuscript that are not due to your comments, one of which is very important (correction of a mistake and making an implicit assumption explicit).

2 Guide to "Revised Manuscript with Track Changes"

In the marked up copy of the revised manuscript, changes are the text parts of the manuscript are denoted by the following means:

- Correction of small typos (spelling, spaces, etc.) are marked in bold blue with a wavy underline. For example, "efficiences" would be corrected as **efficiencies**, "modelassume" would be corrected as **model assume**, etc.
- Deletions are marked in bold red with strikeout like ~~**this is deleted text**~~.
- Additions are marked in bold green with a straight underline like **this is added text**.

Larger typo, grammar, etc. corrections are marked with both deletion and added text.

This marking scheme is not used in the equation environments, which are instead marked via PDF annotations. Additional notes on the changes are in the PDF via additional annotations. Note that these PDF annotations require a suitable viewer and have been tested in Adobe Acrobat, Okular, and Evince. They are likely not visible when using the builtin PDF viewers of web browsers.

3 Response to Comments from Reviewer #1

We will respond to the individual points raised by Reviewer #1 one by one.

3.1 Point 1

> *The level of the English language is overall good, but there are repeated typos all over the manuscript. Please make sure you amend those typos.*

We have gone through and fixed a number of typos and small grammar and clarity issues (added, removed, or replaced words here and there), hopefully all or close to all of them. Small ones are marked in blue bold with a wavy underline, with more major ones marked as deletion (bold red with strikout) and addition (bold green with straight underline).

*lfpn-office@ds.mpg.de

3.2 Point 2

In the Introduction, it is stated that SARS-CoV-2 is an airborne virus. Actually, there is still much debate on that, i.e, to what extent SARS-CoV-2 can be transmitted indirectly at long range. Please add a reference to support this claim.

We have added a few references to the ongoing debate and discussion in the introductory paragraph and made our statements much more conservative in this regard.

3.3 Point 3

The paper deals mostly with the statistical analysis of the phenomenon, in my view overlooking the equally important fluid dynamics behind the generation of the expiratory cloud and aerosolisation. I recommend the Authors to at least mention these important aspects. There is an increasing number of publications available in the literature, see for example:

1 L. Bourouiba, "Turbulent gas clouds and respiratory pathogen emissions. Potential implications for reducing transmission of COVID-19," JAMA Insights 323, 1837 (2020).

2 R. Mittal, R. Ni, and J.-H. Seo, "The flow physics of COVID-19," J. Fluid Mech. 894, F2 (2020).

3. E. Renzi, A. Clarke "Life of a droplet: Buoyant vortex dynamics drives the fate of micro-particle expiratory ejecta," Phys. Fluids 32, 123301 (2020)

To address this as well as Point 1 of Reviewer # 2 which is similar, we have added a new paragraph (5'th paragraph of the Introduction) specifically mentioning these aspects and providing references to them as well as some recent references that cover more than one aspect. In addition to putting some of the existing references to their categories, we added reference 2 suggested above (which is the same as reference 1 suggested by Reviewer #2); references 1, 6, 7, 9, and 10 suggested by Reviewer #2; as well as the following additional references, two of which are a bit older but could be useful to readers.

- Abkarian M, Stone HA. Stretching and break-up of saliva filaments during speech: A route for pathogen aerosolization and its potential mitigation. Physical Review Fluids. 2020;5(10):102301. doi:10.1103/PhysRevFluids.5.102301.
- Bhagat RK, Davies Wykes MS, Dalziel SB, Linden PF. Effects of ventilation on the indoor spread of COVID-19. Journal of Fluid Mechanics. 2020;903:F1. doi:10.1017/jfm.2020.720.
- Bourouiba L, Dehandschoewercker E, Bush JWM. Violent expiratory events: on coughing and sneezing. Journal of Fluid Mechanics. 2014;745:537–563. doi:10.1017/jfm.2014.88.
- Chao CYH, Wan MP, Morawska L, Johnson GR, Ristovski ZD, Hargreaves M, et al. Characterization of expiration air jets and droplet size distributions immediately at the mouth opening. Journal of Aerosol Science. 2009;40(2):122–133. doi:10.1016/j.jaerosci.2008.10.003.

The added references were also useful elsewhere in the manuscript and are referenced as appropriate, such as finding that reference 10 suggested by Reviewer #2 mentions the possible need for a self-proximity aerosol depletion correction for susceptible individuals in the flux limited regime which is more or less, in idea, a local part of $\alpha_{S,f}$ meaning our idea of incorporating it is actually partially predated by Mittal, Meneveau & Wu (2020).

4 Response to Comments from Reviewer #2

We will respond to the individual points raised by Reviewer #2 one by one.

4.1 Point 1

While the introduction discusses some essential research in the field, the reviewer feels the authors should also discuss a few recent developments in the context. Recently the transport and fluid mechanistic aspects of the respiratory droplets have received great attention. For example, the details of the transport, settling, and evaporation of the droplets [1-6]; the effect of speech and plosive sounds on respiratory droplets transport and risk of transmission [7-8]; physics disease transport modeling from droplet dynamics [9-10] have been explored in great details. A summary of these studies could benefit the readers of the current paper since they can add more granularity to the model presented.

[1]. R. Mittal, R. Ni, J.-H. Seo, J. Fluid Mech., 2020, 894, F2.

[2]. T. Dbouk, D. Drikakis, Phys. Fluids, 2020, 32, 053310.

[3]. L. Bourouiba, Jama, 2020, 323(18), 1837

[4]. H. Li, F.Y. Leong, G. Xu, Z. Ge, C. W. Kang, K. H. Lim, Phys. Fluids, 2020, 32(11), 113301.

[5]. S. Chaudhuri, S. Basu, P. Kabi, V. R. Unni, A. Saha, Phys. Fluids, 2020, 32(6), 063309.

[6]. S. Balachandar, S. Zaleski, A. Soldati, G. Ahmadi, L. Bourouiba, Int. J. Multiphase Flow, 2020, 132, 103439

[7]. M. Abkarian, S. Mendez, N. Xue, F. Yang, H. A. Stone, Proc. National Acad, Sc., 2020, 117(41), 25237-25245.

[8]. F. Yang, A. A. Pahlavan, S. Mendez, M. Abkarian, H. A. Stone, Phy. Rev. Fluids, 2020, 5, 122501.

[9]. S. Chaudhuri, S. Basu, A. Saha, Phys. Fluids, 2020, 32(12), 123306.

[10]. R. Mittal, C. Meneveau, W. Wu, Phys. Fluids, 2020, 32, 101903.

To address this as well as Point 3 of Reviewer # 1 which is similar, we have added a new paragraph (5'th paragraph of the Introduction) specifically providing references on these topics by category as well as some recent references that cover more than one aspect (overview references). In addition to putting some of the existing references to their categories, we added references 1 (which is the same as reference 2 suggested by Reviewer #1), 6, 7, 9, and 10 suggested above; ; as well as the following additional references, two of which are a bit older but could be useful to readers.

- Abkarian M, Stone HA. Stretching and break-up of saliva filaments during speech: A route for pathogen aerosolization and its potential mitigation. Physical Review Fluids. 2020;5(10):102301. doi:10.1103/PhysRevFluids.5.102301.
- Bhagat RK, Davies Wykes MS, Dalziel SB, Linden PF. Effects of ventilation on the indoor spread of COVID-19. Journal of Fluid Mechanics. 2020;903:F1. doi:10.1017/jfm.2020.720.
- Bourouiba L, Dehandschoewercker E, Bush JWM. Violent expiratory events: on coughing and sneezing. Journal of Fluid Mechanics. 2014;745:537–563. doi:10.1017/jfm.2014.88.
- Chao CYH, Wan MP, Morawska L, Johnson GR, Ristovski ZD, Hargreaves M, et al. Characterization of expiration air jets and droplet size distributions immediately at the mouth opening. Journal of Aerosol Science. 2009;40(2):122–133. doi:10.1016/j.jaerosci.2008.10.003.

The added references were also useful elsewhere in the manuscript and are referenced as appropriate, such as finding that reference 10 above mentions the possible need for a self-proximity aerosol depletion correction for susceptible individuals in the flux limited regime which is more or less, in idea, a local part of $\alpha_{S,f}$ meaning our idea of incorporating it is actually partially predated by Mittal, Meneveau & Wu (2020).

4.2 Point 2

The Wells-Riley model's primary assumption is that the pathogens (via droplets) are homogeneously distributed in space. However, these pathogens' concentration is expected to be higher close to an infected individual and lower close to a healthy individual. Does the presented model account for such inhomogeneity? If not, can this be included in the as an additional effect?

The model does not account for these inhomogenieties, unfortunately. It in fact assumes that the environment is well-mixed enough and the people spaced sufficiently far enough apart that inhomogeneities are negligible. The good news is that the required corrections would more or less be the same as for conventional single multiplicity Wells-Riley models.

This assumption was discussed a bit in the original submission, but we have added more explicit discussion on this assumption with an explicit statement that the manuscript does not introduce any corrections for such inhomogeneities since they are beyond the scope of this manuscript. We have also added a short qualitative discussion of what partial corrections would look like and that any corrections for single multiplicity Wells-Riley models would work on this model.

5 Changes not Due to Editor and Reviewer Comments

The following changes/additions were made that were not in response to the Editor or Reviewer comments, but we feel are important to make to fix a mistake and problem with the model, update a reference, fix ambiguities, provide a more suitable analytical solution for constant coefficients when $\gamma = 0$, and point readers towards software tools we have developed since the original submission to aid in using the model. We understand that these are more major changes than what the Reviewers suggested.

5.1 Implicit Assumption About Aerosol Nucleation and Fixing A Mistake

In January shortly before receiving the response to our submission, we realized we had implicitly made an assumption in the model in two places, one of them where the assumption is wrong. Specifically, in a sufficiently dry environment where the infectious aerosols will completely dry out in the environment, they must nucleate and make a large discontinuous change in equilibrium diameter if the humidity in the environment increases or if they are inhaled (as an aerosol evaporates, eventually solute saturation is reached after which it will completely evaporate with a large discontinuous jump in equilibrium diameter across the humidity where saturation is reached). In the original submission, we had not thought about nucleation and had implicitly assumed that the completely dried aerosols would nucleate and quickly reach their equilibrium diameter.

When the humidity in the environment raises enough for a dry aerosol to nucleate, the time it takes to grow to the equilibrium diameter (potentially seconds or 10s of seconds) is sufficiently small compared to other time scales that the assumption that $d \approx d_e$ should still be good enough. We now state this assumption explicitly so the reader can be on the lookout for situations where the assumption might pose problems.

But this assumption is an outright mistake for completely dried out aerosols when inhaled since the time scale for growth back to d_0 can exceed the residence time in the respiratory tract. This means that the respiratory tract filtering/absorption efficiency $E_{C,r,j}$ for each person depends on the equilibrium diameter in the respiratory tract (approximately d_0), the diameter in the environment when it is inhaled, and the breathing rate (determines the amount of time the aerosol will be in the respiratory tract); rather than solely on d_0. We have expressed the dependence in terms of d_0, w, and $\lambda_{C,j}$: $E_{C,r,j}(d_0, w(d_0, t), \lambda_{C,j}(t))$. This change has been applied throughout the manuscript as well as adding an explicit discussion of this. We have also directed the reader towards the following references for the nucleation and growth processes as well as the deposition processes in the respiratory tract, which together could be used to construct $E_{C,r,j}(d_0, w(d_0, t), \lambda_{C,j}(t))$.

- Pruppacher HR, Klett JD. Microphysics of Clouds and Precipitation. 2nd ed.
  Dordrecht: Springer Netherlands; 2010.

- ICRP. Human Respiratory Tract Model for Radiological Protection. ICRP Publication 66. Ann. ICRP 24 (1-3). Pergamon Press; 1994. Available from: https://www.icrp.org/publication.asp?id=icrppublication66.

We still make the approximation that $d \approx d_e$ for inhaled aerosols when they leave the respiratory tract (only relevant for the sinks from inhalation $\alpha_{C,f}$ for individuals wearing masks), but now explicitly point this approximation out and discuss when the accuracy of the approximation is not a major issue and when it could be an issue.

Overall, we feel that these additions **MUST** be made to the manuscript since they are correcting a mistake and explicitly stating two other assumptions/approximations that were previously implicit.

5.2 Updating A Reference

We had previously cited the preprint

> Chong KL, Ng CS, Hori N, Yang R, Verzicco R, Lohse D. Extended lifetime of respiratory droplets in a turbulent vapour puff and its implications on airborne disease transmission. medRxiv [Preprint]. 2020;medRxiv 2020.08.04.20168468v1. doi:10.1101/2020.08.04.20168468.

which has now been published. We have updated the reference to it, which is

> Chong KL, Ng CS, Hori N, Yang R, Verzicco R, Lohse D. Extended Lifetime of Respiratory Droplets in a Turbulent Vapor Puff and Its Implications on Airborne Disease Transmission. Physical Review Letters. 2021;126(3):034502. doi:10.1103/PhysRevLett.126.034502.

5.3 Fixing Ambiguity in Binned α_g

In Supplementary Appendix 3 (Binning Diameter), the suggested approximate expression for $\left.\alpha_g\right|_i(t)$ from Eq (7) is ambiguous because it does not specify how to evaluate $w(d_0, T)^2$ over the diameter interval. It has been fixed to suggest taking the average of $w(d_0, T)^2$ over the interval.

5.4 Solutions for Constant Coefficients when $\gamma = 0$

We have added the solutions to the model when the coefficients are constant in time for $\gamma = 0$ for both when $\alpha = 0$ and $\alpha > 0$. These are the solutions for when there is no coupling between the different multiplicities. We have added these solutions explicitly because going through the provided solution in the original manuscript when $\gamma = 0$ is a bit of work due either having to go back to the original ODEs or work through the divisions by zero with limits, and these special cases must be explicitly handled when evaluating the analytical solution in software using hardware accelerated floating point operations.

Not including these special cases in the original manuscript was an oversight. However, we understand if the Editor and/or Reviewers believe the addition should not be included.

5.5 Mentioning and Linking to The Software We Developed for The Model

Since the original submission, we have developed more usable, faster, and more comprehensive software to assist in the model developed in the manuscript (mostly focused on constant coefficients) to replace most of the original non-general *ad hoc* codes we were originally using (a notable exception is the code used for the S5 Appendix which was not replaced). We have released this software to the public to make it easier to use the model developed in our manuscript. We call the software PMADRA (Poly-Multiplicity Airborne Disease Risk Assessment), located at `https://gitlab.gwdg.de/mpids-lfpn-public/pmadra`. This is comprised of

- `libpmadra` (`https://gitlab.gwdg.de/mpids-lfpn-public/pmadra/libpmadra`) Fortran 2008 library for evaluating the analytical solution for constant coefficients once one has α and $\vec{\beta}$, determining the required floating point format for the analytical solution, evaluating the multiplicity corrected exponential model, etc. Version 0.2.1 was used.

- `pypmadra` (`https://gitlab.gwdg.de/mpids-lfpn-public/pmadra/pypmadra`) Python 3.5 or newer package that does the same thing as `libpmadra` (and in fact can use `libpmadra` as an optional accelerator) as well as providing the ability to do the analytical solution for constant coefficients in arbitrary precision floating point as well as evaluating $V_k(\vec{y}, x)$, $U_k(\vec{y}, x)$, and $\vec{n}_\infty$ exactly when α, γ and each element of $\vec{y}$ or $\vec{\beta}$ is rational. Version 0.2.1 was used.

We have updated the figures to using `pypmadra` to the extent possible, and added mention of PMADRA. This provides tools for readers and allows greater scrutiny on our numerical methods.
